# Promoter hypermethylation in male breast cancer: analysis by multiplex ligation-dependent probe amplification

**DOI:** 10.1186/bcr3220

**Published:** 2012-07-05

**Authors:** Robert Kornegoor, Cathy B Moelans, Anoek HJ Verschuur-Maes, Marieke CH Hogenes, Peter C de Bruin, Joost J Oudejans, Paul J van Diest

**Affiliations:** 1 Department of Pathology, University Medical Center Utrecht, Heidelberglaan 100, 3584 CX Utrecht, the Netherlands; 2 Laboratory for Pathology East Netherlands, Burgemeester Edo Bergsmalaan 1, 7512 AD Enschede, the Netherlands; 3 Department of Pathology, St Antonius Hospital, Koekoekslaan 1, 3435 CM Nieuwegein, the Netherlands; 4 Department of Pathology, Diakonessenhuis, Bosboomstraat 1, 3582 KE Utrecht, the Netherlands

## Abstract

**Introduction:**

Epigenetic events are, along with genetic alteration, important in the development and progression of cancer. Promoter hypermethylation causes gene silencing and is thought to be an early event in carcinogenesis. The role of promoter hypermethylation in male breast cancer has not yet been studied.

**Methods:**

In a group of 108 male breast cancers, the methylation status of 25 genes was studied using methylation-specific multiplex ligation-dependent probe amplification. Methylation of more than 15% was regarded indicative for promoter hypermethylation. Methylation status was correlated with clinicopathological features, with patients' outcome and with 28 female breast cancer cases.

**Results:**

Promoter hypermethylation of the genes *MSH6*, *WT1*, *PAX5*, *CDH13*, *GATA5 *and *PAX6 *was seen in more than 50% of the cases, but was uncommon or absent in normal male breast tissue. High overall methylation status was correlated with high grade (*P *= 0.003) and was an independent predictor of poor survival (*P *= 0.048; hazard ratio 2.5). *ESR1 *and *GSTP1 *hypermethylation were associated with high mitotic count (*P *= 0.037 and *P *= 0.002, respectively) and high grade (both *P *= 0.001). No correlation with survival was seen for individual genes. Compared with female breast cancers (logistic regression), promoter hypermethylation was less common in a variety of genes, particularly *ESR1 *(*P *= 0.005), *BRCA1 *(*P *= 0.010) and *BRCA2 *(*P *< 0.001). The most frequently hypermethylated genes (*MSH6*, *CDH13*, *PAX5*, *PAX6 *and *WT1*) were similar for male and female breast cancer.

**Conclusion:**

Promoter hypermethylation is common in male breast cancer and high methylation status correlates with aggressive phenotype and poor survival. *ESR1 *and *GSTP1 *promoter hypermethylation seem to be involved in development and/or progression of high-grade male breast cancer. Although female and male breast cancer share a set of commonly methylated genes, many of the studied genes are less frequently methylated in male breast cancer, pointing towards possible differences between male and female breast carcinogenesis.

## Introduction

Along with genetic alterations, epigenetic events are important in cancer development and progression. Hypermethylation of CpG islands in promoter regions (further denoted methylation) is the most well characterized epigenetic change and is a common mechanism for silencing tumor suppressor genes [1]. Methylation is reversible and therefore is an attractive therapeutic target, and can serve as a marker for therapy response and prognosis [2].

Methylation is very common in virtually all cancer types but can also be a physiological event, as in genomic imprinting [3]. Methylation is involved in the development of female breast cancer, with frequent methylation of *PAX6*, *BRCA2*, *PAX5*, *WT1*, *CDH13 *and *MSH6 *in ductal carcinoma *in situ *and invasive ductal cancer [4]. On the contrary, methylation is less common in estrogen receptor (ER)-negative, lymph node-negative and *BRCA1*-associated female breast cancer [5]. Methylation is thought to be an early event in carcinogenesis of female breast cancer, and the methylation status of specific genes may therefore be useful as a potential screening target in clinical practice [4,6].

Most of the diagnostic and therapeutic algorithms for male breast cancer have been extrapolated from female breast cancer although we and others have already demonstrated that there seem to be important differences between the two. Male breast cancers are more often hormone-positive while *HER2*-amplified and basal-like breast cancers are rare in men [7-10]. Different genes and mechanisms of oncogene activation also play a role in the carcinogenesis of male breast cancer: high-level amplification is less common, but whole chromosome arm gains are more often seen in male breast cancer [11]. Because of its general importance in carcinogenesis, methylation is probably also important in the development of male breast cancer, but this has not yet been studied.

Several techniques are available to assess methylation. The methylation-specific multiplex ligation-dependent probe amplification (MS-MLPA) technique allows simultaneous evaluation of the methylation status of a variety of genes in one PCR reaction. With this high-throughput approach, which shows good correlations with other methylation-specific techniques, a reliable general view of methylation in several important tumor suppressor genes can be obtained [12,13].

In this study we investigated the role of methylation of several important tumor suppressor genes in male breast cancer using MS-MLPA. We correlated methylation patterns with clinicopathological features and prognosis. The results were also compared with a group of female breast cancers.

## Materials and methods

### Patients: specimens and clinical information

One hundred and ten consecutive cases of surgical breast specimens of invasive male breast cancer from 1986 to 2010 were collected from four different pathology laboratories in the Netherlands (St Antonius Hospital Nieuwegein, Diakonessenhuis Utrecht, University Medical Center Utrecht and Laboratory for Pathology East Netherlands) as described in more detail previously [10]. H & E slides were reviewed by three experienced observers (PJvD, RK and AHJV-M) to confirm the diagnosis and to type and grade according to current standards.

Pathology reports were used to retrieve information on age, tumor size and lymph node status. The mean age of these patients was 66 years (range 32 to 89 years). The tumor size ranged from 0.8 to 5.5 cm (average 2.2 cm). In 86% of cases the lymph node status was known, and 55% of these patients had lymph node metastases. The majority of cases were diagnosed (according to the World Health Organization) as invasive ductal carcinoma (90%). The remaining cases were lobular (*n *= 3), mixed type (ductal/lobular) (*n *= 2), invasive cribriform (*n *= 1), papillary (*n *= 1), mucinous (*n *= 2), invasive micropapillary (*n *= 1) or adenoid cystic carcinomas (*n *= 1). According to the modified Bloom and Richardson score [14] most tumors were grade 2 (41%) or grade 3 (36%). Mitotic activity was assessed as described previously [15] with a mean mitotic index per 2 mm^2 ^of 11 (range 0 to 56). For all cases, the hormone receptor and *HER2 *status were reassessed as described previously [10]. Tissue microarray slides were used for immunohistochemical stainings for ER and progesterone receptor (PR), and chromogenic *in situ *hybridization for *HER2 *assessment. Most tumors were ER-positive (102/110, 93%) and PR-positive (71/110; 65%), and *HER2 *amplification was rare (4/110, 4%).

In addition, normal male breast tissue was obtained from 10 autopsies. These patients had no history of a breast tumor. The subareolar region was resected and, after fixation in 4% formalin, was dissected and embedded in paraffin. From these blocks, 4 μm sections were cut and stained for H & E; if ducts were present, the areas richest in ducts were dissected for DNA isolation. Anonymous use of redundant tissue for research purposes is part of the standard treatment agreement with patients in our hospital [16]. Ethical approval was not required.

### DNA extraction and MS-MLPA analysis

Representative tumor areas were identified on H & E-stained slides and corresponding areas (at least 1 cm^2^) were dissected from 8 μm paraffin slides using a scalpel. DNA was extracted by overnight incubation in proteinase K (10 mg/ml; Roche, Almere, the Netherlands) at 56°C. MS-MLPA was performed according to the manufacturers' instructions (MRC Holland, Amsterdam, the Netherlands), using a Veriti^® ^96-well thermal cycler (Applied Biosystems, Foster City, CA, USA). The ME002-B1 kit (MRC Holland), containing 25 tumor suppressor genes (Table 1), was used as previously [4]. Two different CpG probes were available for the genes *MGMT *and *RB1*.

**Table 1:** Genes in the MS-MLPA kit and frequencies of promoter hypermethylation in male breast cancer patients

Gene	Hypermethylation	Chromosome	Gene name
*MSH6 *	104 (96%)	02p16.3	mutS homologue 6
*WT1*	91 (84%)	11p13	Wilms tumor 1
*PAX5*	85 (79%)	09p13.2	Paired box 5
*CDH13*	83 (77%)	16q24.1	Cadherin 13, H-cadherin
*GATA5*	60 (56%)	20q13.33	GATA binding protein 5
*PAX6*	57 (53%)	11p13	Paired box 6
*GSTP1*	47 (44%)	11q13.1	Glutathione S-transferase p1
*THBS1*	21 (19%)	15q14	Thrombospondin 1
*BRCA2*	18 (17%)	13q13.1	Breast cancer gene 2
*CD44*	17 (16%)	11p13	CD44 molecule (Indian blood group)
*TP73 *	14 (13%)	01p36.32	Tumor protein p73
*TP53*	12 (11%)	17p13.1	Tumor protein p53
*ESR1*	9 (8%)	06q25.1	Estrogen receptor 1
*CADM1*	9 (8%)	11q23.2	Cell adhesion molecule 1
*MGMT*	8 (7%)	10q26.3	*O*-6-Methylguanine-DNA methyltransferase
*STK11*	8 (7%)	19p13.3	Serine/threonine kinase 11
*RARB *	5 (5%)	03p24.2	Retinoic acid receptor beta
*PTEN *	5 (5%)	10q23.31	Phosphatase and tensin homologue
*PYCARD*	5 (5%)	16p11.2	PYD and CARD domain containing (TMS1)
*RB1*	3 (3%)	13q14.2	Retinoblastoma 1
*BRCA1*	2 (2%)	17q21.31	Breast cancer gene 1
*CDKN2A *	2 (2%)	09p21.3	Cyclin-dependent kinase inhibitor 2A (p14-ARF)
*VHL *	2 (2%)	03p25.3	von Hippel-Lindau
*ATM*	1 (1%)	11q22.3	Ataxia telangiectasia mutated
*CHFR*	1 (1%)	12q24.33	Checkpoint with forkhead and ring finger domains

Genes contained in the ME002-B1 MS-MLPA kit (MRC Holland, Amsterdam, the Netherlands) and frequencies of promoter hypermethylation (> 15%) in 108 male breast cancer patients.

The principle of MS-MLPA has been described elsewhere in more detail [17]. In short, MS-MLPA kits contain probes for methylation quantification, which are similar to those in conventional multiplex ligation-dependent probe amplification except that the sequence detected by the MS-MLPA probes contains a restriction site for the methylation-sensitive *HhaI *enzyme. After DNA denaturation and overnight incubation with the probe mix, the samples are divided into two tubes, one of which is incubated with *HhaI*. In this tube, unmethylated DNA is digested and not exponentially amplified by PCR. Because methylated DNA is prevented from being digested by *HhaI*, these probes are ligated and therefore amplified by PCR. The ratio between probes incubated with and without *HhaI *gives an estimation of the methylation status. Appropriate negative and positive (Sssi-methylated DNA) controls were taken along with each MS-MLPA run. The PCR products were separated by electrophoresis on an ABI 3730 capillary sequencer (Applied Biosystems).

Methylation analysis was carried out with Genescan v4.1 (Applied Biosystems) and Coffalyser v9.4 (MRC Holland) software. Relative probe peaks were calculated by dividing the signal of each probe by the signal of every reference probe in one sample (intra-sample normalization). For the final methylation status, the ratio of relative probe peaks of the undigested sample (without *HhaI*) and the corresponding digested sample (with *HhaI*) were calculated for each probe. In case two CpG loci were present for one gene (*MGMT *and *RB1*), the mean methylation status was calculated for further analysis.

A promoter methylation ratio > 0.15 (corresponding to > 15% methylation) was regarded as indicative for promoter hypermethylation, based on cell-line experiments and previous experience [4,18]. The cumulative methylation index (CMI) was calculated as the sum of the methylation percentage of all genes [5].

### Comparison with female breast cancer

A previously described group of female breast cancers was used to compare promoter hypermethylation in male and female breast cancer [4]. This group consists of 33 patients with invasive ductal carcinoma and a mean age of 55 years (range 32 to 81 years). The tumor size ranged from 0.5 to 6.5 cm (average 2.1 cm). The mean mitotic activity was 14 per 2 mm^2 ^and, according to the modified Bloom and Richardson score, most tumors were grade 2 (10/33, 30%) or grade 3 (17/33, 52%). ER-positivity was common (27/31, 87%) and 71% of the tumors were PR-positive (22/31, 71%). *HER2 *amplification was seen in two cases (2/31, 6%). The same tumor suppressor kit (ME002-B1; MRC Holland) was used.

### Statistical analysis

Statistical calculations were performed using SPSS for Windows v15.0 (SPSS Inc., Chicago, IL, USA), regarding two-sided *P *< 0.05 as significant. Correlations between promoter hypermethylation (> 15% methylation) and clinicopathological characteristics were calculated with analysis of variance for continuous variables and with the Pearson chi-square test (or Fisher's exact test when appropriate) for categorical variables. The following clinicopathological features were dichotomized: age (> 50 years), tumor size (> 2.0 cm), mitotic count (> 8) and histological grade (1/2 vs. 3). The Mann-Whitney test was used to calculate differences in CMI and clinicopathological features. Correlation between the number of methylated genes and clinicopathological features was calculated with Spearman's rho. Unsupervised hierarchical clustering using the statistical program R [19] was performed to analyze relevant clusters and co-methylation. Absolute methylation percentages were used and all cases with methylation < 5% were pooled together. Logistic regression analysis was performed to compare methylation in male and female breast cancer, taking significant clinicopathological differences between the two groups into account. Backward stepwise method was used until the most predictive variables remained.

Survival data were obtained from the Integral Cancer Registration the Netherlands (IKNL). Outcome data were available for 101 cases with a mean follow-up of 5.7 years. Survival analysis was therefore based on 5-year survival rates. For univariate survival analysis, Kaplan-Meier curves were plotted and analyzed with the log-rank test. Multivariate survival analysis was performed with Cox regression (enter and remove limits 0.10). The CMI and number of methylated genes were dichotomized for survival analysis according to the most predictive threshold.

## Results

### Methylation status by MS-MLPA

In two male breast cancer cases the amount of DNA was insufficient, leaving 108 cases for further analysis. The methylation status of the 25 analyzed tumor suppressor genes is presented in Table 1. All cases except one showed methylation (15% cutoff) of at least one gene, with an average of six genes (range 0 to 26). Methylation was very common for *MSH6 *(96%), *WT1 *(83%), *PAX5 *(79%) and *CDH13 *(77%). On the contrary, methylation was very rare in *RB1 *(3%), *BRCA1 *(2%), *CDKN2A *(2%), *VHL *(2%), *ATM *(1%) and *CHFR *(1%). The mean CMI was 364 (range 129 to 904).

In male breast tissue derived from autopsies, gynecomastia was seen in three cases. The other seven cases harbored normal male breast ducts. Methylation was seen in the genes *MSH6 *(4/10, 40%), *ESR1 *(2/10, 20%), *PAX5 *(1/10, 10%) and *CDH13 *(1/10, 10%). No methylation was found in all of the other genes. The mean CMI in these cases was also low at 16 (range 11 to 27).

### Correlation with clinicopathological features

Higher CMI was correlated with high mitotic count (*P *= 0.046) and high grade (*P *= 0.003). The number of methylated genes was significantly higher in grade 3 cancers (*P *= 0.034), and correlated with a high mean mitotic count (*P *= 0.021). Two individual genes were associated with a more aggressive phenotype: the mean mitotic count was higher in tumors with *ESR1 *(10 vs. 16; *P *= 0.037) and *GSTP1 *(8 vs. 14; *P *= 0.002) methylation. Both genes were also associated with high grade (both *P *= 0.001). For *ESR1 *eight out of nine methylated tumors were grade 3, and for *GSTP1 *25 out of 47 methylated tumors were grade 3. Finally, tumors with *MGMT *methylation had a mean tumor size of 3.2 cm, which was significantly larger compared with tumors without *MGMT *methylation (2.1 cm; *P *= 0.002). No association was seen between any genes on the one hand and age, lymph node, PR and *HER2 *status on the other.

### Cluster analysis

**Figure 1:**
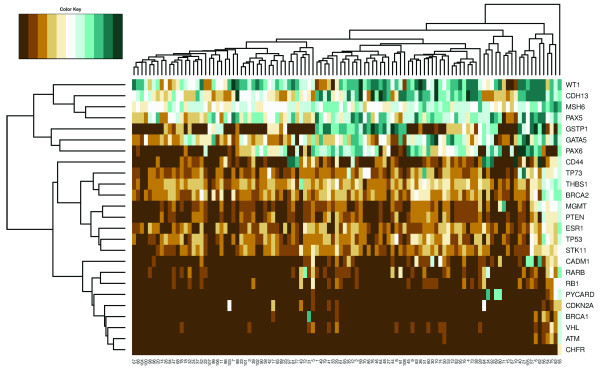
**Hierarchical clustering analysis of male breast cancer patients**. Unsupervised hierarchical clustering of absolute methylation percentages in 25 genes in 108 male breast cancer patients. One gene cluster consisted of *WT1*, *CDH13*, *MSH6*, *PAX5*, *GSTP1*, *GATA5 *and *PAX6*, seven genes in which methylation was very common. The second cluster was formed by genes with intermediate methylation rates (5 to 19%), and in the third cluster the remaining genes with little methylation (< 8%) grouped together. No clear distinctive clusters of male breast cancer cases were found.

Hierarchical cluster analysis revealed three groups of clustered genes (Figure 1). One group consisted of the genes *WT1*, *CDH13*, *MSH6*, *PAX5*, *GSTP1*, *GATA5 *and *PAX6*, seven genes in which methylation was very common. Indeed, in 15% of cases all these genes showed methylation. The second cluster was formed by genes with intermediate methylation rates (5 to 19%). In the third group the remaining genes clustered together. Methylation was rare (< 8%) in these genes. Regarding all of the patients, male breast cancer cases were not divided into clear distinctive clusters. At least four different groups could be identified and these clusters displayed no distinct clinicopathological features. One case did not fit into any of the groups. This grade 3 male breast cancer case showed a high methylation ratio in nearly all genes.

### Comparison with female breast cancer

Because breast cancer is a heterogeneous disease, only luminal-type male breast cancer and luminal-type female breast cancer (defined by ER and/or PR expression) were compared. In this approach, age was the only clinicopathological feature that was significantly different between the two groups. Male breast cancer patients were significantly older (66 years vs. 54 years; *P *< 0.001).

**Figure 2:**
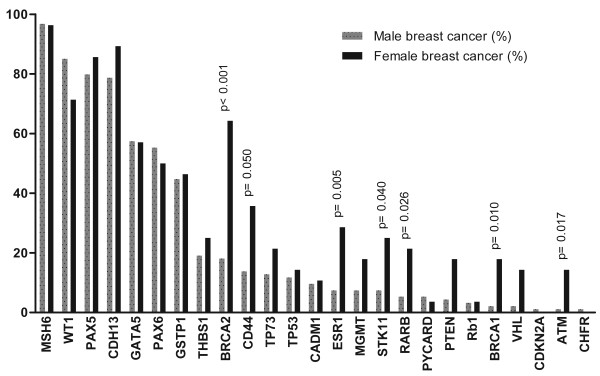
**Methylation status of the 25 studied genes in luminal-type breast cancer**. Promoter hypermethylation (> 15% methylation) of the 25 studied genes in luminal-type male (*n *= 95) and luminal-type female (*n *= 28) breast cancer.

Figure 2 illustrates the methylation status of the 25 studied genes in luminal-type male (*n *= 95) and luminal-type female (*n *= 28) breast cancer. Methylation was much less frequent in male breast cancer in a variety of genes. Particularly, *ESR1*, *BRCA1 *and *BRCA2 *were less often methylated compared with female breast cancer and were strong independent predictors of gender in logistic regression analysis (*P *= 0.005, *P *= 0.010 and *P *< 0.001, respectively). The genes *CD44 *(*P *= 0.050), *RARB *(*P *= 0.026), *ATM *(*P *= 0.017) and *STK11 *(*P *= 0.040) also showed less frequent methylation in male breast cancer. On the other hand, the high frequency of methylation in *MSH6*, *PAX5*, *PAX6 *and *CDH13 *was shared between male and female breast cancer.

Only age was taken into account during logistic regression analysis using gender as the determinant, because no other clinicopathological feature was significantly different between the two groups. When leaving out age and using the Pearson chi-square test, methylation in *PTEN *and *VHL *was also significantly less common in male breast cancer (*P *= 0.029 and *P *= 0.025, respectively). None of the studied genes was more frequently methylated in male breast cancer.

### Survival analysis

**Figure 3:**
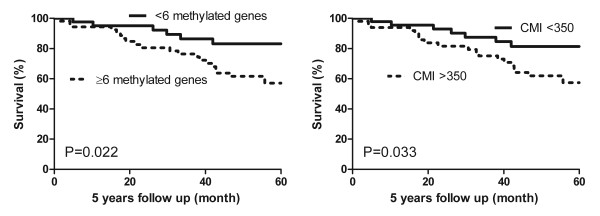
**Five-year survival according to number of methylated genes and cumulative methylation index**. Five-year survival with corresponding *P *values (log rank) according to high number of methylated genes (≥ 6) and high cumulative methylation index (CMI > 350).

Grade 3 (*P *= 0.027), high mitotic count (> 8; *P *= 0.015) and large tumor size (> 2.0 cm; *P *= 0.036) were correlated with decreased 5-year survival as expected. No individual methylated gene was significantly correlated with patients' outcome, although tumors with *GATA5 *methylation showed a trend towards decreased 5-year survival (64% vs. 82%; *P *= 0.083). When the number of methylated genes was dichotomized using a threshold of six methylated genes, however, the group with six or more methylated genes had significantly decreased survival compared with tumors with less than six methylated genes (*P *= 0.022; Figure 3), but was not a significant independent prognostic factor in Cox regression (*P *= 0.057). Tumors with high CMI (> 350) also had decreased survival (*P *= 0.033; Figure 3) and high CMI was an independent prognosticator in Cox regression (*P *= 0.048; hazard ratio 2.5).

## Discussion

Promoter hypermethylation is an important gene-silencing mechanism thought to be an early event in carcinogenesis [1]. Understanding the epigenetic role in male breast cancer is important to gain further insight into male breast carcinogenesis and for the identification of potential biomarkers for diagnosis and treatment [20,21]. Epigenetic changes in male breast cancer had not yet been studied and therefore we investigated promoter hypermethylation in a large group of 108 patients with this rare disease using the high-throughput MS-MLPA approach, enabling evaluation of the methylation status of a variety of genes in one PCR.

Not surprisingly, methylation does occur in male breast cancer. The genes *MSH6*, *WT1*, *PAX5*, *CDH13*, *GATA5 *and *PAX6 *showed promoter hypermethylation in more than 50% of cases, indicating that these genes are probably often involved in male breast carcinogenesis. These genes are required for normal development of several organ systems and/or play a role in DNA repair, cell adhesion, cell growth and migration, although the function of some of these genes is still poorly understood [22-26]. Loss of function of both alleles leads to complete knockdown of these genes, which may facilitate malignant transformation. Methylation, with aberrant silencing of one of these alleles, could be the initiating event, the second hit or both [27]. *MSH6 *methylation was also quite common in the normal male breast, although at a lower frequency than our group of male breast cancer cases. The other commonly methylated genes in male breast cancer were not found to be methylated in our 10 cases of normal male breast tissue, confirming the important role of methylation in the development of male breast cancer.

In male breast cancer, methylation was very rare in *BRCA1*, *CDKN2A*, *VHL*, *ATM *and *CHFR *(< 2%) - indicating that methylation of these genes does not seem to play a prominent role in male breast carcinogenesis.

Male breast cancer with an aggressive phenotype harbored an increased number of methylated genes and had a higher CMI. In addition, tumors with six or more methylated genes or high CMI had a worse outcome. High CMI was even an independent predictor of poor survival when corrected for grade, mitotic count and tumor size. This indicates that accumulation of methylated genes and an overall higher methylation status seem to be important in the development of more aggressive male breast cancer with poor survival. The hallmark of high-grade breast cancer is genetic instability [28], which in male breast cancer seems to include accumulation of methylated genes. A similar trend was noted in female breast cancer, and female breast cancer patients with an increasing number of methylated genes also have an unfavorable outcome [4,29,30].

Two single genes were identified in which methylation was correlated with high mitotic count and high grade: *ESR1 *and *GSTP1*. High-grade breast cancer is believed to arise from high-grade precursor lesions by gaining different genetic and epigenetic changes compared with low-grade breast cancer [31,32]. *ESR1 *and *GSTP1 *methylation could be important in the development of these high-grade male breast cancers. *GSTP1 *belongs to a family of metabolic enzymes and is involved in the detoxification of carcinogens and chemotherapeutic agents by conjugating them with glutathione [33]. In female breast cancer, *GSTP1 *hypermethylation is correlated with high-grade ductal carcinoma *in situ *and high-grade invasive breast cancer, presence of lymph node metastasis and poor outcome [4,30,34,35]. ER, encoded by *ESR1*, is an important factor in breast cancer, because studies in females have shown that patients with hormone-negative tumors do not benefit from endocrine therapy [36]. In the present study we could not demonstrate a relation between *ESR1 *methylation and ER expression, although this needs to be interpreted with caution since only seven out of 108 cases were ER-negative in the present study. Another recent study also concluded that the relation between *ESR1 *methylation and protein expression is weak and unlikely to represent a predominant mechanism of ER silencing [37]. There was also no relation between methylation and expression of TWIST as shown by us, so this may not be unusual [38]. Larger series of ER-negative male breast cancer cases will be needed to further explore this relationship. Similar to female breast cancer, methylation of *ESR1 *seems to be a biomarker for high malignant male breast cancer. Indeed, in female breast cancer *ESR1 *promoter hypermethylation has been correlated with poor prognosis [39]. *ESR1 *methylation and *GSTP1 *methylation were not significantly correlated with poor survival in our group of male breast cancer and therefore do not seem to be useful prognostic biomarkers in male breast cancer.

Compared with female breast cancer, methylation was less common in male breast cancer in several of the studied genes, particularly *ESR1*, *BRCA1 *and *BRCA2*. *BRCA1 *and *BRCA2 *promoter hypermethylation was encountered in, respectively, 2% and 18% of the male breast cancers, but was seen in 18% and 64% of the female breast cancers, using the same approach and similar cutoff criteria. These results points towards possible important differences between female and male breast carcinogenesis with regard to methylation. *BRCA1 *methylation is more common in relatively young, premenopausal women [40], which could explain the higher incidence in female breast cancer since the male breast cancer patients were significantly older than the female breast cancer patients. However, in the present study we corrected for age in logistic regression, so gender-specific differences also seem to play a role here. Differences in genetic predisposition may also influence the epigenetic profile of these tumors and could be responsible for some of the differences found in promoter hypermethylation between male and female breast cancer. Approximately 10% of men with breast cancer are known to have a genetic predisposition, and especially *BRCA2 *mutations seem to be important [41]. Unfortunately no data regarding *BRCA *germline mutations were available for both cohorts, but it seems probable that there is a higher rate of hidden *BRCA2 *mutation carriers in the male breast cancer group. This higher rate of *BRCA2 *mutation carriers may well explain the lower rate of *BRCA2 *promoter hypermethylation in the male breast cancer group compared with female breast cancers [42]. Interestingly, genes with frequent methylation in male breast cancer (*MSH6*, *CDH13*, *PAX5*, *PAX6 *and *WT1*) were also very commonly methylated in female breast cancer.

The methylation status of both groups was obtained using the same technique. However, the male breast cancer cases were microdissected by a scalpel and the female breast cancer cases by laser microdissection. Although the latter method is more precise we do not think this may have influenced our results. The male breast cancer tumors were quite large and rich in tumor cells and could therefore be well harvested for DNA isolation based on scalpel dissection. Besides, multiplex ligation-dependent probe amplification is relatively insensitive to tumor cell content [43].

## Conclusion

Methylation seems to be important in the development of male breast cancer. More than 50% of the tumors showed methylation in *MSH6*, *WT1*, *PAX5*, *CDH13*, *GATA5 *and *PAX6*. The accumulation of methylated genes and an overall high methylation status was correlated with a more aggressive phenotype and poor survival. *ESR1 *and *GSTP1 *were the only single genes associated with mitotically active and high-grade male breast cancers. Compared with female breast cancer, methylation occurred less often in male breast cancer. On the other hand, the most frequently methylated genes were shared between male and female breast cancer. Our results point towards differences in carcinogenesis between male and female breast cancer, hidden behind similarities.

## Abbreviations

ATM: ataxia telangiectasia mutated; BRCA1: breast cancer gene 1; BRCA2: breast cancer gene 2; CADM1: cell adhesion molecule 1; CDH13: cadherin 1; CDKN2A: cyclin-dependent kinase inhibitor 2A; CHFR: checkpoint with forkhead and ring finger domains; CMI: cumulative methylation index; ER: estrogen receptor; ESR1: estrogen receptor 1; GATA5: GATA binding protein 5; GSTP1: glutathione S-transferase p1; H & E: hematoxylin and eosin; HER2: human epidermal growth receptor 2; MGMT: *O*-6-methylguanine-DNA methyltransferase; MSH6: mutS homologue 6; MS-MLPA: methylation-specific multiplex ligation-dependent probe amplification; PAX5: paired box 5; PAX6: paired box 6; PCR: polymerase chain reaction; PR: progesterone receptor; PTEN: phosphatase and tensin homologue; PYCARD: PYD and CARD domain containing; RARB: retinoic acid receptor beta; RB1: retinoblastoma 1; STK11: serine/threonine kinase 11; THBS1: thrombospondin 1; TP53: tumor protein p53; TP73: tumor protein p73; VHL: von Hippel-Lindau; WT1: Wilms tumor 1.

## Competing interests

The authors declare that they have no competing interests.

## Authors' contributions

PJvD, RK and AHJV-M conceived the experiments. RK, MCHH, JJO and PCdB were involved in collecting male breast cancer cases. RK and CBM carried out the experiments and analyzed data. All authors were involved in writing the paper and had final approval of the submitted and published versions.
